# Laceration of the Anterior Vaginal Wall and Its Repair

**Published:** 1891-09

**Authors:** T. J. Watkins

**Affiliations:** Chicago, Ill.


VOL. VIII. SEPTEMBER, 1891. NO. 7.

TO CONTRIBUTORS.

Articles for publication must reach this office not later than 15th inst.; and are expected to be published exclusively in this JOURNAL. Extra copies of JOURNAL furnished contributors when requested. Reprints by special arrangement. Publication of an article does not necessarily imply endorsement of the views therein expressed.

ATLANTA MEDICAL AND SURGICAL JOURNAL, Post-office box 431.

### Original Communications.

### LACERATION OF THE ANTERIOR VAGINAL WALL AND ITS REPAIR.*

BY T. J. WATKINS, M. D., CHICAGO.

Laceration of the anterior vaginal wall has hitherto received little attention. Emmet ** and Schatz † have considered this subject only in its relation to rupture of the levator ani muscle, and state that its repair is impracticable. Mundé ‡ reports a case of median separation accompanied by hernia of the bladder.

Byford § is, so far as I have been able to ascertain from a somewhat extensive study of the literature, the only author who

*Read before the Gynecological Society of Chicago, April 17th, 1891.

**"Principles and Practice of Gynecology," page 264.

†Centralblatt fur Gynakologie, No. 40, 1883.

‡American Journal of Obstetrics, June, 1890, page 614.

§"The Practice of Medicine and Surgery applied to the Diseases and Accidents Incident to Women," fourth edition, pages 173, 479, and 501.

has appreciated in any degree the true nature of the lesion, and the only operator who has suggested a rational method for its repair.

In the consideration of lacerations of the pelvic floor, all the authorities, so far as I have been able to determine, have considered only the rupture of its muscles. It is unphysiological to attribute continuous support to muscles, therefore the connective tissue alone remains to be considered. The connective tissue of the anterior vaginal wall forms a tense, firm band across the vagina opposite the neck of the bladder, which becomes gradually thinner as it approaches the uterus and as it extends along the urethra. It is attached to the bony pelvis on either side, and its reticular arrangement is such that it permits much more longitudinal than transverse freedom of motion—that is, it is so arranged as to give elastic support to the uterus, and to prevent prolapse of the urethra and bladder. The tension which this band gives to the vagina is apparent to the touch ; and on introducing a Sims speculum, with the patient in the left lateral position, the effect upon the anterior vaginal wall can be easily seen, that is, from the introitus vaginæ to the uterus the anterior vaginal wall presents :

1. A convexity corresponding to the urethral curve.
2. A marked concavity opposite the trigone of the bladder.
3. A straight line or a slight convexity from this point to the uterus.

When this fascia is intact and involuted, urethrocele and cystocele cannot occur. The prevailing theory that urethrocele and cystocele are dependent upon and cannot occur without laceration of the posterior vaginal wall is erroneous, because—

1. Extensive laceration of the posterior vaginal wall even through the sphincter ani, frequently occurs without urethrocele or cystocele.

2. Urethrocele and cystocele occur without laceration of the posterior vaginal wall.

3. Incision of the posterior vaginal wall—that is, artificial laceration—never produces urethrocele or cystocele.

This time-honored fallacy may be explained by the fact that both walls of the vagina are often simultaneously ruptured, and that the posterior rupture is much more apparent than the anterior.

Laceration of the anterior vaginal wall may be either unilateral or bilateral. I have never met with a case of median laceration, and have been able to find only one case on record.* The lesion is usually submucous, and occurs at or near the insertion of the fascia into the bony pelvis. It often deprives the horizontal rami of the pubes of their fascial covering for a variable distance from the urethra, and may involve the levator ani muscle, as mentioned by Emmet and Schatz.† The location and extent of the laceration are easily detected by touch, and verified by inspection of the abnormal curvature of the anterior vaginal wall. The amount of the urethrocele and cystocele which result is entirely dependent upon the extent and location of the laceration, and upon the amount of involution which has taken place.

*Etiology.*—The child's head, in its passage through the parturient canal, may produce laceration of the anterior vaginal wall—

1. By the tension and pressure incident to the engagement of the vesico-vaginal septum between it and the pubes.

2. By tearing and grinding of the connective tissue from its attachment.

Schatz† mentions anterior laceration of the levator ani muscle by instruments, and advises against oblique application of forceps.

*Symptomatology.*—The objective symptoms have already been considered. The subjective symptoms, which are dependent upon the amount of urethrocele and cystocele, are—

1. Partial incontinence of urine. The urine escapes upon exertion, such as coughing, sneezing, laughing, walking, lifting, or as soon as the desire to urinate is experienced.

2. Total incontinence of urine.

The other subjective symptoms are those which are described

---

*Munde, Op. cit.

†Op. cit.

in the text-books in the consideration of cystocele and prolapse of the uterus.

*Diagnosis.*—The diagnosis depends upon the recognition of the local lesion and of the resultant symptoms.

*Treatment.*—I. Prophylaxis. The prophylactic treatment consists—

1. In the support of the vesico-vaginal septum while the fœtal head is entering the true pelvis—that is, the prevention of the engagement of the vesico-vaginal septum between the head and the pubes.

2. In the prevention of excessive pressure of the head upon the pubic arch (Schatz).

3. In the employment of the usual measures for hastening involution.

II. Operation. The rational operative treatment is to restore, as far as possible, the lacerated fascia to its normal condition. The usual operations on the anterior vaginal wall have failed to accomplish this result, because—

1. They roll together tissues not involved in the laceration.

2. They include so little connective tissue that, as a rule, no permanent support is obtained.

3. Retroposition of the uterus frequently follows.

4. They produce little or no effect upon the urethrocele.

The multiplicity of median operations on the anterior vaginal wall would seem to indicate that the results of these operations have been more or less unsatisfactory.

An operation to be rational—

1. Must be upon the portion of the anterior vaginal wall which has been torn—that is, it must bring together, as far as possible, the lacerated tissues.

2. Must include much of the pelvic fascia of the anterior vaginal wall.

3. Must neither shorten the anterior vaginal wall nor bring the lateral walls of the vagina together in front of the uterus.

The unsatisfactory result of the median operation induced me to attempt a lateral operation which I have performed twenty times, and which has in every case practically fulfilled the indications.* The technique is as follows:

The patient being placed in the left lateral position, the anterior vaginal wall is exposed by Sims' speculum, and a point to the side of the urethra, near its meatus, caught by a tenaculum. The denudation is commenced at this point, and extends along the antero-lateral walls of the vagina to a point beyond the prolapse. This point may be opposite the neck of the bladder, or the denudation may extend even as far as the lateral aspect of the cervix uteri. The breadth of the denuded surface is dependent upon the extent of the urethrocele and cystocele; that is, it should be sufficiently wide to take in all the redundant tissues of the urethrocele and cystocele. The denudation may be upon one or both sides, according as the laceration is unilateral or bilateral. Should the denuded surface extend beyond the neck of the bladder, the cervix uteri should be drawn firmly upward and backward while the sutures are being inserted and tied. For this purpose I have adopted the method recommended by Dr. E. C. Dudley, in the technique of Emmet's anterior colpororrhaphy†, of fastening the cervix uteri to the end of the speculum by means of a suture.

Beginning at the uterine end of the denudation, buried silk-worm-gut sutures are now passed from side to side in a curved line which has its convexity directed outward and forward. Each suture as inserted is tied, and traction is exerted toward the cervix while the next suture is being introduced and tied. The sutures should include as much connective tissue as possible, care being taken not to injure the bladder, ureters, or urethra. After passing the base of the trigone of the bladder, the sutures should be passed deeply into the lateral wall so as to include the fascia of the posterior vaginal wall near its insertion into the pubes, and as deeply into the anterior vaginal wall as the increased thickness of the vesico-vaginal septum from this point outward will permit. The fixation suture should now be re-

---

* Dr. John A. Lyons, of this city, has performed this operation three times with results practically identical with my own.

† Pepper's "System of Medicine," page 162.

moved without making traction on the cervix. The ends of the sutures should be left long and should be turned into the vagina.

The after-treatment consists in the measures usually employed in plastic operations upon the vagina. The use of the catheter should, if possible, be avoided. The stitches may be removed after a week, or may be allowed to remain for two or three weeks, according to the requirements of the individual case.

The operation has entirely fulfilled both the mechanical and symptomatic indications, except in one case, in which, on account of suppuration around some of the sutures, only partial relief was obtained. Up to this time, so far as I have been able to ascertain, the results of the operation have been permanent.

I append a tabular statement of the twenty cases in which I have performed this operation, and of the three cases operated upon by Dr. Lyons.

See table on following page.

